# 2-(Methoxy­meth­yl)adamantan-2-yl 2-methyl­acrylate

**DOI:** 10.1107/S1600536809042639

**Published:** 2009-10-23

**Authors:** Qingwei Meng, Yueqing Li, Pingping Chen, Weijie Zhao, Jinzong Yang

**Affiliations:** a State Key Laboratory of Fine Chemicals, Dalian Unversity of Technology, PO Box 90, Zhongshan Road 158, Dalian 116012, People’s Republic of China

## Abstract

The title compound, C_16_H_24_O_3_, has a cage-type mol­ecular structure and is of inter­est with respect to its photochemical properties. The structure displays non-classical inter­molecular C—H⋯O hydrogen bonding, which links the mol­ecules into a three-dimensional network.

## Related literature

For the synthesis of the title compound and its analogues, see: Hui *et al.* (2007 ▶); Isobe *et al.* (2007 ▶); Kikugawa (2009 ▶); Sasaki *et al.* (2007 ▶); Takahashi *et al.* (2006 ▶). For related photoresist preparations, see: Chen *et al.* (2009 ▶); Fedynyshyn (2009 ▶); Okago *et al.* (2009 ▶); Padmanaban *et al.* (2009 ▶); Yoo *et al.* (2009 ▶).
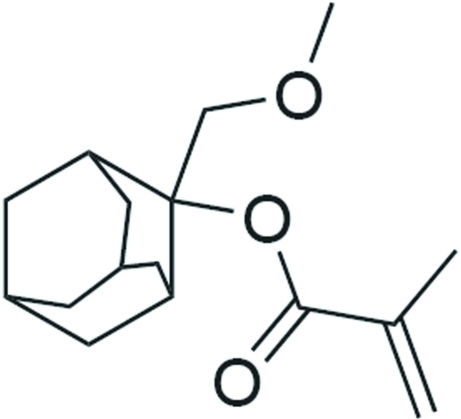


## Experimental

### 

#### Crystal data

C_16_H_24_O_3_
*M* *_r_* = 264.35
Monoclinic, 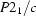

*a* = 14.1385 (12) Å
*b* = 7.5265 (7) Å
*c* = 13.9712 (12) Å
β = 102.461 (6)°
*V* = 1451.7 (2) Å^3^
*Z* = 4
Mo *K*α radiation
μ = 0.08 mm^−1^
*T* = 298 K
0.40 × 0.35 × 0.30 mm

#### Data collection

Bruker SMART APEXII CCD diffractometer
Absorption correction: none
9914 measured reflections
3701 independent reflections
2096 reflections with *I* > 2σ(*I*)
*R*_int_ = 0.025

#### Refinement

*R*[*F* ^2^ > 2σ(*F* ^2^)] = 0.047
*wR*(*F* ^2^) = 0.159
*S* = 1.03
3701 reflections
173 parameters
H-atom parameters constrained
Δρ_max_ = 0.15 e Å^−3^
Δρ_min_ = −0.13 e Å^−3^

### 

Data collection: *APEX2* (Bruker, 2005 ▶); cell refinement: *SAINT-Plus* (Bruker, 2001 ▶); data reduction: *SAINT-Plus*; program(s) used to solve structure: *SHELXS97* (Sheldrick, 2008 ▶); program(s) used to refine structure: *SHELXL97* (Sheldrick, 2008 ▶); molecular graphics: *SHELXTL* (Sheldrick, 2008 ▶); software used to prepare material for publication: *SHELXTL*.

## Supplementary Material

Crystal structure: contains datablocks I, global. DOI: 10.1107/S1600536809042639/rk2167sup1.cif

Structure factors: contains datablocks I. DOI: 10.1107/S1600536809042639/rk2167Isup2.hkl

Additional supplementary materials:  crystallographic information; 3D view; checkCIF report

## Figures and Tables

**Table 1:** Hydrogen-bond geometry (Å, °)

*D*—H⋯*A*	*D*—H	H⋯*A*	*D*⋯*A*	*D*—H⋯*A*
C16—H16*A*⋯O2^i^	0.93	2.58	3.499 (2)	171

Symmetry code: (i) 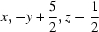.

**Fig. 1.**
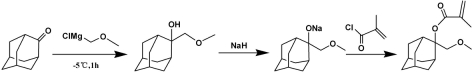
The synthesis path of title compound.

**Fig. 2.**
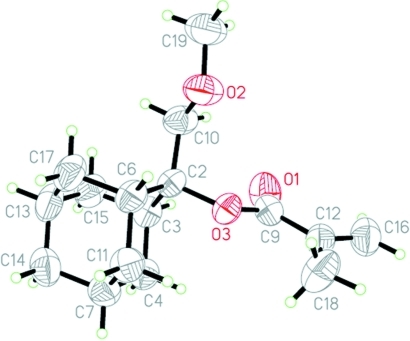
The molecular structure of title compound with the atom-numbering scheme. Displacement ellipsoids are drawn at the 50% probability level. H atoms are presented as a small spheres of arbitrary radius.

**Fig. 3.**
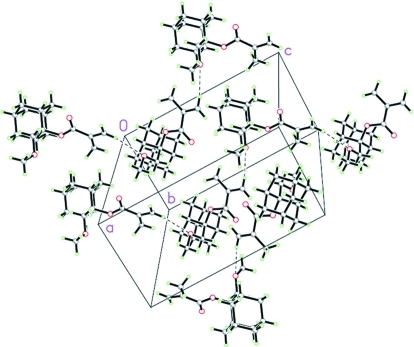
Molecular packing in the crystal. Hydrogen bonds are shown as dashed lines.

## supplementary crystallographic information

### Comment

The photoresist is the key material for the preparation of integrated circuit plates. With the development of integrated circuit, the quest for high performance of the photoresist is changing. From 1993 till now, 193 nm photoresist is always being a research hot spot. As the important monomers of polymer matrix for 193 nm photoresist, adamant-2-yl methacrylates are potential and the design of such compounds has received significant attention (Chen *et al.*, 2009; Fedynyshyn, 2009; Hui *et al.*, 2007; Isobe *et al.*, 2007; Kikugawa, 2009; Okago *et al.*, 2009; Padmanaban *et al.*, 2009; Sasaki *et al.*, 2007; Takahashi *et al.*, 2006; Yoo *et al.* 2009).

As a part of studying the effect of side chain substitution on the structures of adamant-2-yl methacrylates, the crystal structure of 2-methyl-acrylic acid 2-methoxymethyl-adamantan-2-yl ester has been determined. The title compound is prepared *via* three steps including Grignard reaction, etherafication and esterification (Fig. 1). The conformation of the C═O and C═C bonds of the methacrylic group are *syn* to each other but not coplanar (Fig.2). The torsion angle O1–C9–C12═C16 is equal to 9.4 (3)°. The geometry of the molecule as well as 1.1996 (19)Å, 1.478 (2)Å and 1.340 (2)Å distances of O1–C9, C9–C12 and C12═C16 bonds, indicate no obvious delocalization of the electron pairs of C═O and C═C within the methacrylic group. The non-classical C16–H16A···O2^i^ intermolecular hydrogen bonds link the molecules into a three-dimensional network (Table 1, Fig. 3). Symmetry code (i):  *x*, -*y*+5/2, *z*-1/2.

### Experimental

The synthesis of title compound was shown in Fig.1. The crude product was recrystalized by petroleum ether in the yield of 60%. ^1^H-NMR (CDCl_3_, 400 MHz): 1.58-2.53 (14*H*, m), 1.95 (3*H*, s), 3.35 (3*H*, s), 4.08 (2*H*, s), 5.52 (1*H*, s), 6.10 (1*H*, s); Elemental analysis (%) Calcd (Found): C: 72.25 (72.69), H: 9.16 (9.15), O: 18.40 (18.16).

### Refinement

All H atoms attached to C atoms were treated as riding, with C–H = 0.9700Å for ethylene group, with C–H = 0.9700Å for methylene group, C–H = 0.9800Å for methyne group and C–H = 0.9600Å for methyl group with *U*_iso_(H) = 1.2*U*_eq_(C) of the carrier atoms to which they are attached and *U*_iso_(H) = 1.5*U*_eq_(C) for the methyl groups.

### Crystal data

**Table:**

C_16_H_24_O_3_	*F*(000) = 576
*M**_r_* = 264.35	*D*_x_ = 1.210 Mg m^−^^3^
Monoclinic, *P*2_1_/*c*	Melting point: 318 K
Hall symbol: -P 2ybc	Mo *K*α radiation, λ = 0.71073 Å
*a* = 14.1385 (12) Å	Cell parameters from 2653 reflections
*b* = 7.5265 (7) Å	θ = 3.0–25.0°
*c* = 13.9712 (12) Å	µ = 0.08 mm^−^^1^
β = 102.461 (6)°	*T* = 298 K
*V* = 1451.7 (2) Å^3^	Block, colourless
*Z* = 4	0.40 × 0.35 × 0.30 mm

### Data collection

**Table:**

Bruker SMART APEXII CCD diffractometer	2096 reflections with *I* > 2σ(*I*)
Radiation source: fine-focus sealed tube	*R*_int_ = 0.025
graphite	θ_max_ = 28.7°, θ_min_ = 1.5°
φ and ω scans	*h* = −19→18
9914 measured reflections	*k* = −6→10
3701 independent reflections	*l* = −18→18

### Refinement

**Table:**

Refinement on *F*^2^	Secondary atom site location: difference Fourier map
Least-squares matrix: full	Hydrogen site location: inferred from neighbouring sites
*R*[*F*^2^ > 2σ(*F*^2^)] = 0.047	H-atom parameters constrained
*wR*(*F*^2^) = 0.159	*w* = 1/[σ^2^(*F*_o_^2^) + (0.0765*P*)^2^ + 0.0779*P*] where *P* = (*F*_o_^2^ + 2*F*_c_^2^)/3
*S* = 1.02	(Δ/σ)_max_ < 0.001
3701 reflections	Δρ_max_ = 0.15 e Å^−^^3^
173 parameters	Δρ_min_ = −0.13 e Å^−^^3^
0 restraints	Extinction correction: *SHELXL97* (Sheldrick, 2008), Fc^*^=kFc[1+0.001xFc^2^λ^3^/sin(2θ)]^-1/4^
Primary atom site location: structure-invariant direct methods	Extinction coefficient: 0.036 (4)

### Special details

**Table:**

Geometry. All s.u.'s (except the s.u. in the dihedral angle between two l.s. planes) are estimated using the full covariance matrix. The cell s.u.'s are taken into account individually in the estimation of s.u.'s in distances, angles and torsion angles; correlations between s.u.'s in cell parameters are only used when they are defined by crystal symmetry. An approximate (isotropic) treatment of cell s.u.'s is used for estimating s.u.'s involving l.s. planes.
Refinement. Refinement of *F*^2^ against ALL reflections. The weighted *R*-factor *wR* and goodness of fit *S* are based on *F*^2^, conventional *R*-factors *R* are based on *F*, with *F* set to zero for negative *F*^2^. The threshold expression of *F*^2^ > σ(*F*^2^) is used only for calculating *R*-factors(gt) *etc*. and is not relevant to the choice of reflections for refinement. *R*-factors based on *F*^2^ are statistically about twice as large as those based on *F*, and *R*-factors based on ALL data will be even larger.

### Fractional atomic coordinates and isotropic or equivalent isotropic displacement parameters (Å^2^)

**Table:**

	*x*	*y*	*z*	*U*_iso_*/*U*_eq_	
O3	0.72029 (7)	1.00159 (13)	0.70066 (7)	0.0492 (3)	
C2	0.73378 (10)	0.86448 (19)	0.77704 (10)	0.0475 (4)	
C3	0.78456 (12)	0.7003 (2)	0.74679 (11)	0.0523 (4)	
H3A	0.7419	0.6415	0.6912	0.063*	
C4	0.87843 (11)	0.7558 (2)	0.71813 (11)	0.0564 (4)	
H4A	0.9096	0.6523	0.6974	0.068*	
H4B	0.8641	0.8383	0.6636	0.068*	
O2	0.59138 (8)	0.97066 (16)	0.82779 (9)	0.0729 (4)	
C6	0.80243 (11)	0.9541 (2)	0.86316 (11)	0.0530 (4)	
H6A	0.7713	1.0604	0.8827	0.064*	
C7	0.94636 (12)	0.8433 (2)	0.80430 (11)	0.0614 (5)	
H7A	1.0064	0.8781	0.7851	0.074*	
O1	0.63265 (9)	0.83342 (17)	0.58041 (9)	0.0746 (4)	
C9	0.67070 (11)	0.9716 (2)	0.60916 (12)	0.0518 (4)	
C10	0.63535 (12)	0.8197 (2)	0.79806 (13)	0.0612 (5)	
H10A	0.5939	0.7708	0.7395	0.073*	
H10B	0.6432	0.7303	0.8491	0.073*	
C11	0.89683 (12)	1.0069 (2)	0.83410 (12)	0.0593 (5)	
H11A	0.8831	1.0902	0.7799	0.071*	
H11B	0.9395	1.0649	0.8889	0.071*	
C12	0.66997 (12)	1.1315 (2)	0.54756 (11)	0.0563 (4)	
C13	0.87610 (14)	0.6618 (3)	0.91968 (12)	0.0724 (6)	
H13A	0.8909	0.5792	0.9751	0.087*	
C14	0.96933 (13)	0.7145 (3)	0.88993 (13)	0.0743 (6)	
H14A	1.0009	0.6096	0.8712	0.089*	
H14B	1.0130	0.7701	0.9448	0.089*	
C15	0.80964 (14)	0.5713 (2)	0.83353 (13)	0.0682 (5)	
H15A	0.7507	0.5332	0.8525	0.082*	
H15B	0.8414	0.4670	0.8144	0.082*	
C16	0.61220 (14)	1.1289 (3)	0.45791 (13)	0.0762 (6)	
H16A	0.6088	1.2277	0.4173	0.091*	
H16B	0.5755	1.0285	0.4363	0.091*	
C17	0.82633 (15)	0.8258 (3)	0.94946 (12)	0.0694 (5)	
H17A	0.8686	0.8835	1.0046	0.083*	
H17B	0.7673	0.7913	0.9693	0.083*	
C18	0.72945 (16)	1.2843 (3)	0.58613 (15)	0.0865 (6)	
H18A	0.7206	1.3767	0.5376	0.130*	
H18B	0.7964	1.2498	0.6023	0.130*	
H18C	0.7105	1.3273	0.6439	0.130*	
C19	0.50466 (14)	0.9249 (3)	0.85691 (17)	0.0884 (7)	
H19A	0.4757	1.0301	0.8769	0.133*	
H19B	0.5189	0.8428	0.9107	0.133*	
H19C	0.4605	0.8709	0.8029	0.133*	

### Atomic displacement parameters (Å^2^)

**Table:**

	*U*^11^	*U*^22^	*U*^33^	*U*^12^	*U*^13^	*U*^23^
O3	0.0590 (6)	0.0445 (6)	0.0440 (6)	−0.0045 (5)	0.0108 (5)	0.0001 (5)
C2	0.0569 (9)	0.0424 (9)	0.0452 (8)	−0.0030 (7)	0.0152 (7)	0.0032 (6)
C3	0.0641 (9)	0.0441 (9)	0.0484 (9)	0.0007 (7)	0.0116 (7)	−0.0037 (7)
C4	0.0652 (10)	0.0590 (10)	0.0477 (9)	0.0097 (8)	0.0180 (8)	−0.0025 (7)
O2	0.0659 (8)	0.0589 (8)	0.1038 (10)	0.0058 (6)	0.0402 (7)	0.0083 (7)
C6	0.0657 (10)	0.0538 (10)	0.0410 (8)	0.0004 (8)	0.0148 (7)	−0.0064 (7)
C7	0.0559 (10)	0.0743 (12)	0.0550 (10)	0.0008 (8)	0.0138 (8)	−0.0043 (8)
O1	0.0801 (9)	0.0617 (8)	0.0703 (8)	−0.0072 (6)	−0.0098 (6)	−0.0078 (6)
C9	0.0494 (9)	0.0534 (10)	0.0508 (9)	0.0042 (8)	0.0069 (7)	−0.0043 (8)
C10	0.0634 (10)	0.0510 (10)	0.0750 (11)	−0.0036 (8)	0.0278 (9)	0.0027 (8)
C11	0.0613 (10)	0.0633 (11)	0.0509 (9)	−0.0100 (8)	0.0070 (8)	−0.0099 (8)
C12	0.0599 (10)	0.0617 (11)	0.0497 (9)	0.0121 (8)	0.0171 (8)	0.0040 (8)
C13	0.0928 (14)	0.0767 (13)	0.0481 (10)	0.0222 (11)	0.0158 (10)	0.0158 (9)
C14	0.0721 (12)	0.0890 (14)	0.0571 (10)	0.0170 (10)	0.0038 (9)	−0.0038 (10)
C15	0.0838 (12)	0.0502 (10)	0.0742 (12)	0.0096 (9)	0.0252 (10)	0.0105 (9)
C16	0.0827 (13)	0.0820 (14)	0.0611 (11)	0.0171 (11)	0.0097 (10)	0.0073 (10)
C17	0.0880 (13)	0.0784 (13)	0.0442 (9)	0.0110 (10)	0.0197 (9)	0.0033 (9)
C18	0.1140 (17)	0.0687 (13)	0.0771 (13)	−0.0163 (12)	0.0210 (12)	0.0151 (11)
C19	0.0695 (12)	0.0876 (15)	0.1194 (17)	0.0119 (11)	0.0458 (12)	0.0176 (13)

### Geometric parameters (Å, °)

**Table:**

O3—C9	1.3379 (19)	C11—H11A	0.9700
O3—C2	1.4671 (17)	C11—H11B	0.9700
C2—C10	1.521 (2)	C12—C16	1.340 (2)
C2—C6	1.530 (2)	C12—C18	1.458 (2)
C2—C3	1.534 (2)	C13—C14	1.518 (3)
C3—C4	1.525 (2)	C13—C15	1.519 (3)
C3—C15	1.534 (2)	C13—C17	1.523 (2)
C3—H3A	0.9800	C13—H13A	0.9800
C4—C7	1.518 (2)	C14—H14A	0.9700
C4—H4A	0.9700	C14—H14B	0.9700
C4—H4B	0.9700	C15—H15A	0.9700
O2—C10	1.4004 (19)	C15—H15B	0.9700
O2—C19	1.4150 (19)	C16—H16A	0.9300
C6—C17	1.524 (2)	C16—H16B	0.9300
C6—C11	1.529 (2)	C17—H17A	0.9700
C6—H6A	0.9800	C17—H17B	0.9700
C7—C11	1.519 (2)	C18—H18A	0.9600
C7—C14	1.520 (2)	C18—H18B	0.9600
C7—H7A	0.9800	C18—H18C	0.9600
O1—C9	1.1996 (19)	C19—H19A	0.9600
C9—C12	1.478 (2)	C19—H19B	0.9600
C10—H10A	0.9700	C19—H19C	0.9600
C10—H10B	0.9700		
			
C9—O3—C2	122.37 (12)	C6—C11—H11B	109.7
O3—C2—C10	108.39 (12)	H11A—C11—H11B	108.2
O3—C2—C6	102.87 (11)	C16—C12—C18	122.91 (18)
C10—C2—C6	113.42 (12)	C16—C12—C9	117.33 (17)
O3—C2—C3	111.18 (11)	C18—C12—C9	119.75 (15)
C10—C2—C3	112.16 (13)	C14—C13—C15	108.99 (13)
C6—C2—C3	108.46 (12)	C14—C13—C17	110.06 (17)
C4—C3—C2	109.71 (13)	C15—C13—C17	109.69 (15)
C4—C3—C15	108.37 (13)	C14—C13—H13A	109.4
C2—C3—C15	109.55 (12)	C15—C13—H13A	109.4
C4—C3—H3A	109.7	C17—C13—H13A	109.4
C2—C3—H3A	109.7	C13—C14—C7	109.36 (14)
C15—C3—H3A	109.7	C13—C14—H14A	109.8
C7—C4—C3	110.37 (12)	C7—C14—H14A	109.8
C7—C4—H4A	109.6	C13—C14—H14B	109.8
C3—C4—H4A	109.6	C7—C14—H14B	109.8
C7—C4—H4B	109.6	H14A—C14—H14B	108.3
C3—C4—H4B	109.6	C13—C15—C3	109.86 (15)
H4A—C4—H4B	108.1	C13—C15—H15A	109.7
C10—O2—C19	110.83 (14)	C3—C15—H15A	109.7
C17—C6—C11	108.51 (14)	C13—C15—H15B	109.7
C17—C6—C2	109.73 (14)	C3—C15—H15B	109.7
C11—C6—C2	110.36 (11)	H15A—C15—H15B	108.2
C17—C6—H6A	109.4	C12—C16—H16A	120.0
C11—C6—H6A	109.4	C12—C16—H16B	120.0
C2—C6—H6A	109.4	H16A—C16—H16B	120.0
C4—C7—C11	108.58 (13)	C13—C17—C6	109.51 (12)
C4—C7—C14	109.81 (15)	C13—C17—H17A	109.8
C11—C7—C14	109.53 (13)	C6—C17—H17A	109.8
C4—C7—H7A	109.6	C13—C17—H17B	109.8
C11—C7—H7A	109.6	C6—C17—H17B	109.8
C14—C7—H7A	109.6	H17A—C17—H17B	108.2
O1—C9—O3	124.81 (15)	C12—C18—H18A	109.5
O1—C9—C12	124.37 (16)	C12—C18—H18B	109.5
O3—C9—C12	110.82 (14)	H18A—C18—H18B	109.5
O2—C10—C2	111.15 (13)	C12—C18—H18C	109.5
O2—C10—H10A	109.4	H18A—C18—H18C	109.5
C2—C10—H10A	109.4	H18B—C18—H18C	109.5
O2—C10—H10B	109.4	O2—C19—H19A	109.5
C2—C10—H10B	109.4	O2—C19—H19B	109.5
H10A—C10—H10B	108.0	H19A—C19—H19B	109.5
C7—C11—C6	110.02 (14)	O2—C19—H19C	109.5
C7—C11—H11A	109.7	H19A—C19—H19C	109.5
C6—C11—H11A	109.7	H19B—C19—H19C	109.5
C7—C11—H11B	109.7		

### Hydrogen-bond geometry (Å, °)

**Table:**

*D*—H···*A*	*D*—H	H···*A*	*D*···*A*	*D*—H···*A*
C16—H16A···O2^i^	0.93	2.58	3.499 (2)	171

Symmetry codes: (i) *x*, −*y*+5/2, *z*−1/2.
